# The Role of Hydrogen Sulfide in Respiratory Diseases

**DOI:** 10.3390/biom11050682

**Published:** 2021-05-01

**Authors:** Saadullah Khattak, Qian-Qian Zhang, Muhammad Sarfraz, Pir Muhammad, Ebenezeri Erasto Ngowi, Nazeer Hussain Khan, Saqib Rauf, Yi-Zhen Wang, Hui-Wen Qi, Di Wang, Attia Afzal, Xin-Ying Ji, Dong-Dong Wu

**Affiliations:** 1 Henan International Joint Laboratory for Nuclear Protein Regulation, School of Basic Medical Sciences, Henan University, Kaifeng 475004, China; saadullah@henu.edu.cn (S.K.); 13323950805@163.com (Q.-Q.Z.); chiefpharm@gmail.com (M.S.); ebenezerngowi92@gmail.com (E.E.N.); Kakakhan3514@gmail.com (N.H.K.); yizhanwang713@163.com (Y.-Z.W.); qihuiwen0314@163.com (H.-W.Q.); w15858109412@163.com (D.W.); 2 School of Life Sciences, Henan University, Kaifeng 475004, China; 3 Faculty of Pharmacy, The University of Lahore, Lahore 56400, Pakistan; 4 Henan-Macquarie University Joint Centre for Biomedical Innovation, School of Life Sciences, Henan University, Kaifeng 475004, China; pir.muhammad786@gmil.com; 5 Department of Biological Sciences, Faculty of Science, Dar es Salaam University College of Education, Dar es Salaam 2329, Tanzania; 6 Department of Zoology, Islamia College University Peshawar, Peshawar, Khyber Pakhtunkhwa 25120, Pakistan; saqibrauf8@gmail.com; 7 Kaifeng Key Laboratory of Infection and Biological Safety, School of Basic Medical Sciences, Henan University, Kaifeng 475004, China; 8 School of Stomatology, Henan University, Kaifeng 475004, China

**Keywords:** hydrogen sulfide, respiratory diseases, metabolism processes, signaling pathways

## Abstract

Respiratory diseases are leading causes of death and disability around the globe, with a diverse range of health problems. Treatment of respiratory diseases and infections has been verified to be thought-provoking because of the increasing incidence and mortality rate. Hydrogen sulfide (H_2_S) is one of the recognized gaseous transmitters involved in an extensive range of cellular functions, and physiological and pathological processes in a variety of diseases, including respiratory diseases. Recently, the therapeutic potential of H_2_S for respiratory diseases has been widely investigated. H_2_S plays a vital therapeutic role in obstructive respiratory disease, pulmonary fibrosis, emphysema, pancreatic inflammatory/respiratory lung injury, pulmonary inflammation, bronchial asthma and bronchiectasis. Although the therapeutic role of H_2_S has been extensively studied in various respiratory diseases, a concrete literature review will have an extraordinary impact on future therapeutics. This review provides a comprehensive overview of the effective role of H_2_S in respiratory diseases. Besides, we also summarized H_2_S production in the lung and its metabolism processes in respiratory diseases.

## 1. Introduction

Respiratory diseases are generally the most common disorders, mainly characterized by lesions located in the trachea, bronchi, lungs and chest. Typical clinical symptoms include cough, asthma and chest pain. Severe cases involve difficulty in breathing or even respiratory failure. Overall, respiratory diseases are physiologically categorized as obstructive or restrictive [1]. Obstructive diseases usually inhibit the flow rate into and out of the lungs, while the obstructive circumstances cause a reduction in the functional lung volume. Common respiratory diseases are pneumo-thorax, pulmonary bulla, emphysema, lung cancer, pulmonary heart disease, respiratory failure, pulmonary embolism, lung abscess, pneumonia, neonatal pneumonia, pediatric pneumonia, bronchitis, asthma, tuberculosis, pneumoconiosis and interstitial lung disease. Moreover, the respiratory tract infections can further be discriminated by the location (i.e., upper or lower tract infections) affected by bacterial or viral infections [2,3]. Over the past three decades, respiratory diseases’ incidence has increased progressively in developed countries and attracted much attention. The World Health Organization (WHO) has defined chronic respiratory diseases as one of the chief diseases smiting the human world and has drawn much attention to their prevention, identification and treatment [4,5,6].

Hydrogen sulfide (H_2_S) is found in a gaseous state, is soluble in water, and has a distinctive odor like rotten eggs. Formerly, H_2_S was understood to be toxic due to its respiratory complex activities in mitochondria, resulting in cellular incapability to metabolize oxygen in an oxidative manner [7,8,9]. However, in recent years, there has been increasing evidence that H_2_S plays an essential role in various physiological and pathological processes, such as inflammation [10,11,12,13], neuromodulation [14], injury repair [15] and hypertension [16]. Furthermore, Szabo et al. threw light on cancer, disclosing that cystathionine beta-synthase (CBS), one of the critical enzymes involved in the formation of H_2_S, is highly expressed in colorectal cancer cells in comparison with nearby adjacent normal mucosal margin cells [17]. On the other hand, both shRNA silencing and pharmacological treatment-mediated CBS inhibition can induce the suppression of the multiplication of cancerous cells in the colon both in vitro and in vivo.

**Figure 1:**
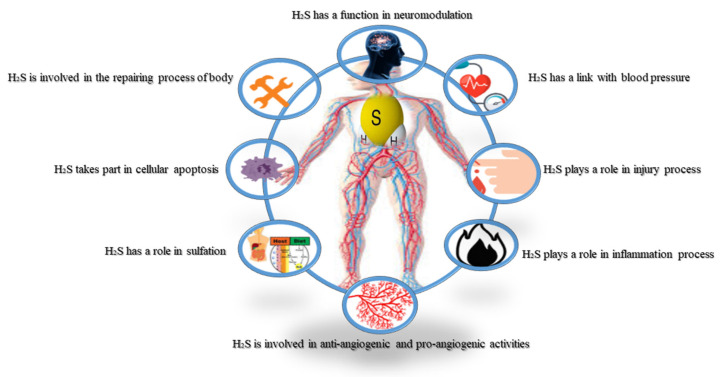
A schematic diagram showing the roles of H_2_S in human physiology and pathology. H_2_S is formed throughout the body and moderates signaling processes in various tissues, including neuromodulation, blood pressure, injury, inflammation, anti-angiogenesis, pro-angiogenesis and sulfhydration apoptosis repair processes of the human body (H_2_S: hydrogen sulfide).

Furthermore, both gene silencing of CBS and the pharmacological inhibition of CBS cause distinct energy conversion at the cell level in cancerous colon cells [17]. The ratio of CBS to H_2_S plays a vital role in cancer progression, including in ovarian cancer [18] and breast cancer [19]. Otherwise, cystathionine γ-lyase (CSE) overexpression, another H_2_S-producing enzyme, has also been reported in melanoma [20]. Moreover, H_2_S plays a vital role in cellular activities such as proliferation, angiogenesis, the function of the mitochondria and vascular relaxation, and is thought to be an essential factor in cancer biology [21,22,23,24,25,26,27]. H_2_S is reported to induce both suppressive and inhibitory effects in human aorta SMC via the ERK/EGFR/MMP-2 and Akt/PTEN signaling pathways, and H_2_S activates MAPK and caspase-3 to initiate apoptosis [28]. Meanwhile, sulfhydration of NF-κB by H_2_S promotes anti-apoptotic activities [29]. It has been reported that phosphorylation of protein kinase Akt by H_2_S can also induce anti-angiogenic properties [30,31,32]. The antioxidant capacity of H_2_S has been investigated in specific animal experiments [33,34]. Nevertheless, the mechanisms by which H_2_S is produced inside tumor cells and enhances cancer cell growth, are still being unraveled. Figure 1 highlights the essential roles of H_2_S in the human body.

H_2_S is closely related to respiratory activities and can affect the outcome of various respiratory diseases. For example, several studies have indicated that serum levels of H_2_S in patients with chronic obstructive pulmonary disease (COPD) are low. This event is correlated with reduced chronic inflammation of the airway and vascular remodeling. Such activity shows the curative effects on pulmonary hypertension and asthma [35,36,37,38]. H_2_S in the respiratory tract induces anti-apoptosis and anti-inflammatory effects and regulates vascular permeability. According to recent reports, patients with acute exacerbation of COPD have significantly low serum H_2_S, while serum levels of H_2_S in smokers are much lower than in non-smokers [39].

Several pathways describe the damage of H_2_S in respiratory diseases. However, some mechanisms are still not fully clarified. H_2_S inhibits respiratory rhythm in neonates through the medulla [40]. H_2_S can reduce other substances and is oxidized via circulating oxidants. H_2_S, together with NO, CO and cyanide, are highly toxic, and micromolar concentrations can ultimately inhibit mitochondrial respiration [7]. H_2_S is found in combination with sulfate and sulfur species. The compounds have variable forms ranging from persulfide and polysulfide to elemental sulfur. The compounds are reactive [41]. A species of sulfane-sulfur acts as a store of H_2_S, which maintains toxicity and allows H_2_S to react with biological signals through sulfhydration [42,43]. Similarly, the sulfur compounds act through S-sulfhydration and are involved in most activities of H_2_S [44]. Altered biosynthesis of H_2_S is also linked with sulfate-sulfur levels based on pathophysiology, suggesting a close relationship.

The defective production of H_2_S initiates several systemic disorders, and such a situation reveals the advance of effective pharmacological mediators that increase H_2_S levels. H_2_S modulation in pharmacy is a recent dynamic field that is well-reported and examined for specific significance [45,46,47]. Currently, a considerable number of natural and artificial compounds have been documented as potent H_2_S donors [48], and many of them are in clinical trials for the treatment of cardiovascular disease (SG-1002 for heart failure) [48] and cancer (sulforaphane) [49]. In this review, the production and metabolism of H_2_S in the lung are highlighted. Besides, the mechanisms and roles associated with the effects of H_2_S in respiratory diseases are further explored.

## 2. Regulation of H_2_S and H_2_S-Mediated Sulfhydration in the Lung

**Figure 2:**
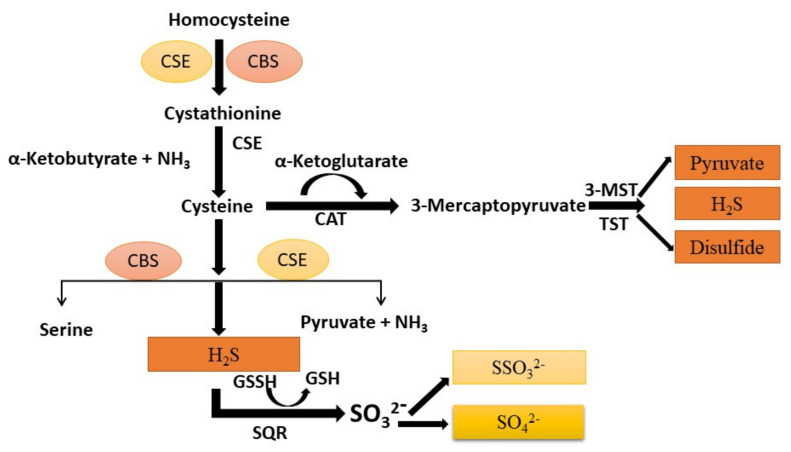
Illustration of vascular synthesis of H_2_S formed by a catalytic process in several enzymes (CSE, CBS, and 3-MSPT) in the lung. Cysteine is generated from homocysteine through transculturation pathways intervened by CBS and CSE. H_2_S forms from homocysteine and cysteine via CBS and CSE. 3-MSPT forms 3-MST-cysteine persulfide (MST-SSH) using mercapto pyruvate, which is formed from cysteine via CAT. H_2_S is formed from MST-SSH via a non-enzymatic reaction. H_2_S is oxidized via sulfide oxidation to form thiosulfate and sulfate. H_2_S is produced from thiosulfate through a non-enzymatic reaction through reductants via the catalytic activity of thiosulfate sulfurtransferase or 3-MST. H_2_S: hydrogen sulfide; SQR: sulfide-quinone reductase; CBS: cystathionine beta-synthase; CSE: cystathionine γ-lyase; 3-MPST: 3-mercaptopyravute sulfurtransferase; TST: thiosulfate sulfurtransferase; CAT: cysteine aminotransferase; GSSH: glutathione.

H_2_S is produced via CSE, CBS and 3-mercaptopyruate transferase (3-MPST) in the lung, but various species or cell types show different expression levels of the three enzymes. Their catalytic activities are reinforced by the reducing enzymes, mainly sulfide-quinone reductase (SQR) and thiosulfate sulfide or thiocyanate (TST) as shown in Figure 2 [50].

The CSE is localized in the endothelium and smooth muscle, while 3-MPST is present in the mitochondria and cytoplasm [51]. Both human airway smooth muscle cells (SMCs) and human lung primary fibroblast MRC-5 cells express CSE and CBS. Immunohistochemical staining shows that CSE is present in the peripheral lung tissues of the airway and pulmonary vessels in rat lung, and mutually CSE and CBS are primarily expressed in pulmonary blood vessels, SMCs and endothelial cells, and airway SMCs in mouse lungs [52]. The catabolic process of H_2_S in the mitochondria is thiosulfate production, which can be further converted to sulfide and then sulfate by rhodanese enzyme action. Besides, methylation of H_2_S by thiol S-methyl transferase can produce dimethyl sulfide.

Most of the cellular responses mediated by H_2_S initiate after sulfhydration and post-translational modification of proteins [53]. Persulfides of H_2_S can modify proteins containing the thiol group. The mechanism through which H_2_S targets a particular thiol protein for S-sulfhydration is in its infancy. Some experimental data suggest that H_2_S attacks thiol-containing proteins, which oxidise as thiolate ions for S-sulfhydration. For example, cysteine residues with a low pKa exist as thiolate anions in typical situations and hence are more definitely confronted by numerous oxidants and are susceptible to S-sulfhydration [54]. The acid–base idea might offer a latent clarification of the mechanism of protein S-sulfhydration. The S-sulfhydration of tissue/cell-specific proteins may occur due to the altered enzymatic activity of H_2_S-producing proteins.

## 3. The Role of H_2_S in Respiratory Diseases

### 3.1. H_2_S and Chronic Obstructive Pulmonary Diseases

Pulmonary diseases such as emphysema, COPD and chronic bronchitis affect the respiratory tract’s airflow. The hindrance is inferred in developing common chronic diseases such as respiratory failure, and pulmonary and heart diseases [55]. One of the key preventable causes of COPD is smoking. However, the mechanism of COPD is not entirely understood. Generally, COPD results from airway inflammation interacting with reactive oxygen species (ROS) [39]. Evidence shows that serum H_2_S levels are significantly reduced in patients with COPD with acute exacerbations. Wang et al. show that H_2_S acts efficiently to improve respiration and reduce histopathological variations, such as lung edema and permeability.

Oxidative stress, inflammation and airway remodeling also decrease via H_2_S treatment [56]. H_2_S exerts both pro- and anti-inflammatory effects. H_2_S is anti-inflammatory and cyto-protective due to its ability to act as an antioxidant and a reducing agent, and its scavenging features [57]. NaHS, a donor of H_2_S, inhibits the in vitro production of intracellular oxidation and cellular damage induced by nitrates, hypochlorous acid and nitrous oxide (NO). It also inhibits the activity and expression of nicotinamide adenine dinucleotide phosphate (NADPH) and scavenges lipid peroxide [58]. Oleic acid induced lung injury in an animal model, while NaHS inoculation reduced lung injuries and plasma levels of interleukin (IL)-6 and IL-8, and the accumulation of inflammatory cells [59,60]. Moreover, in a murine model, the decrease in pro-inflammatory cytokine IL-1β and the rise of the anti-inflammatory cytokine IL-10 occurred after the administration of H_2_S in smoke and burn-induced lung injury [61].

Furthermore, the elevation in the expression of CSE, CBS and H_2_S levels in the pancreas, lung, liver, kidney and plasma of both mice and rats could cause acute inflammation [62]. The smoke of cigarettes is a primary etiological factor for the development of COPD in rat lungs. Treatment with NaHS can reduce lung inflammation and airway resistance caused by smoke [63]. A study shows that inhaled H_2_S develops lung function and prevents bronchial hyper-reactivity by moderating mast cells and fibroblast initiation [64]. Higher levels of serum H_2_S were positively correlated with severe COPD in stable COPD patients. Conversely, H_2_S levels in the serum are decreased in exacerbated COPD in a steady disease state [37].

In contrast, H_2_S levels in the sputum are higher in exacerbated COPD than in steady-state COPD, non-smokers and healthy subjects [65]. NaHS also protects against oxidative stress, airway inflammation, remodeling and an enhanced development rate of emphysema induced by tobacco smoke [39]. H_2_S formation offers a new mechanism for suppressing airway smooth muscle (ASM) cell propagation and cytokine release. H_2_S donors inhibit propagation and cytokine release in COPD ASM cells by inhibiting CBS and 3-MPST. However, COPD ASM cells’ capacity to react to H_2_S donors is not significant in smoker and non-smoker cells [66]. H_2_S treatment inhibited elevated levels of transforming growth factor-beta 1 (TGF-β1) and Smad in a cigarette smoke-induced COPD model via the inhibition of TGF-β1 and Smad pathways.

**Figure 3:**
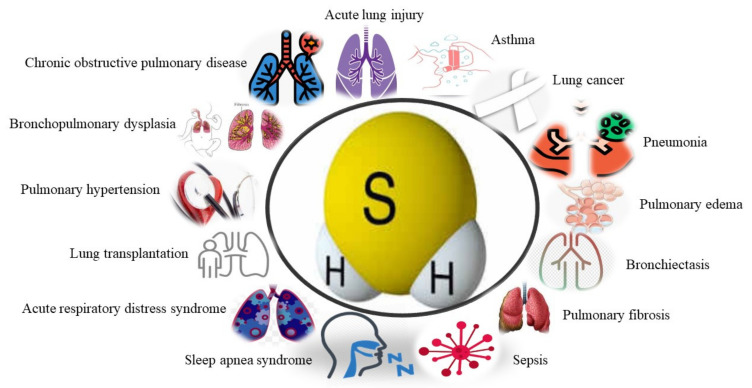
The roles of H_2_S in human respiratory diseases, including COPD, ALI, asthma, lung cancer, pneumonia, pulmonary edema, bronchiectasis, pulmonary fibrosis, sepsis, SAS, ARDS, lung transplantation, pulmonary hypertension and bronchopulmonary dysplasia. COPD: chronic obstructive pulmonary disease; ALI: acute lung injury; ARDS: acute respiratory distress syndrome; SAS: sleep apnea syndrome; BPD: bronchopulmonary dysplasia.

It has been revealed that the serum levels of H_2_S in smoker subjects are much lower than in non-smokers [39]. The endogenous H_2_S is associated with the activity and severity of COPD [37]. Further studies showed that H_2_S could protect macrophages from exposure to inflammation and oxidative stress, thereby enhancing macrophages’ corticosteroid sensitivity [67]. Besides, low levels of H_2_S in exhaled gases can be used to predict eosinophilia in patients [68]. Moreover, the sputum-to-serum ratio of H_2_S can be used to predict obstructive neutrophilic inflammation and COPD progression [68]. The imbalance of H_2_S/Hcy may contribute to COPD pathogenesis combined with cardiovascular diseases, providing a new target for treatment [69]. The intrinsic enzymatic mechanism of H_2_S expression in human airway SMCs has shown the potential for H_2_S being exploited to treat obstructive pulmonary disease (Figure 3) [70].

### 3.2. H_2_S and Acute Lung Injury (ALI)

ALI is considered to be a set of medical symptoms, such as hikes in the permeability of the epithelial and pulmonary vascular system, acute inflammation and microvascular damage, leading to acute respiratory failure and respiratory distress syndrome [65]. Many clinical diseases can cause ALI, such as pancreatic inflammatory lung injury, ventilator lung injury and burn lung injury [71,72].

H_2_S reduces lung injury through numerous signaling pathways [73,74]. H_2_S also assisted in reducing oxidative stress and inflammation to control LPS-induced acute ALI [75]. Exogenous H_2_S prevented ALI by reducing mitochondrial lipid peroxidation and attenuating pro-inflammatory responses positively related to the H_2_S dose to protect the cell structure in LPS-induced rat models [76]. H_2_S inhalation prevents ALI by regulating p38 MAPK signal transduction and Nox-2 expression and synergistic inhibition of ROS formation [77]. Treating rats with H_2_S reduced the transcription of iNOS mRNA, iNOS and nitric oxide (NO); inhibited the activation of NF-κB p65 and attenuated oxidative stress, thereby preventing ALI [78]. H_2_S significantly reduces inflammation and pulmonary edema by regulating the TLR-4-Myd88-NF-κB pathway and AQP-1/AQP-5 expression [79]. Another study showed that thiosulfate inhibits NF-ҝB signaling in the pulmonary vascular endothelium to prevent ALI [80]. Simultaneously, the inhalation of H_2_S triggers genes for anti-apoptosis and anti-inflammation via the regulation of activating transcription factor-3 (ATF-3), demonstrating that ATF3 is noticeably involved in H_2_S-mediated protection [81]. Moreover, it has been revealed that dexamethasone can activate the PI3K pathway to improve the induction of H_2_S in developing acute ALI by increasing the expression of claudin-5 [82]. Similarly, NaHS treatment inhibits the inflammation and oxidation reactions via activation of Nrf-2 cell signaling in preventing lung injury after explosive limb trauma [83]. Furthermore, it has been shown that H_2_S also plays a role in preventing damage escalation in the alveoli and pulmonary hypertension (PHT) for lung injury induced by O_2_ [84]. While in another study, it was reported that intraperitoneal injection of 1 mg/kg NaHS improved the pulmonary levels of H_2_S and oxidative stress-related signs (ROS, myeloperoxidase (MPO) and malondialdehyde (MDA)) in a time-dependent way. Liu et al. pointed out that H_2_S attenuates oleic acid (OA)-induced lung injury by protective and upregulated endoplasmic reticulum proteins [85]. H_2_S also protects against ALI by reducing the expression of MMP-2 and MMP-9 [86]. Some studies reported that H_2_S induces a low metabolic status in anesthetized rats and prevents ventilator-induced lung injury by reducing lung inflammation unrelated to body temperature [87]. H_2_S can also reduce lung L/R injury pressure by reducing lung oxidation [15]. Alternatively, a study showed that an increment in the phosphorylation of myosin light chain (MLC) is protective against the toxicity of NaHS at the cellular level [33]. Meanwhile, an elevation in SMC properties, for instance, the expression of transgelin and motility, or a decrease in actomyosin improves cell survival after exposure to NaHS [87].

#### 3.2.1. Pancreatic Inflammatory Lung Injury

Pancreatic inflammatory lung injury encompasses a set of inflammatory diseases, such as acute pancreatitis. Severe acute pancreatitis is dangerous with poor prognosis, high mortality and early multiple organ failure, especially ALI. After acute pancreatitis, the H_2_S synthase enzyme CSE in the pancreas induces morphological lung changes due to alveolar thickening and inflammatory cell infiltration [10]. Up to one third of all pancreatitis patients develop ALI or acute respiratory distress syndrome (ARDS), accounting for 60% of pancreatitis-associated deaths [88]. Bhatia et al. reported a good correlation between the level of H_2_S and the severity of pancreatitis, and indicated that the pro-inflammatory effects of H_2_S might be mediated by chemokines [89]. Tamizhselvi suggested that H_2_S may exhibit potent vasodilation activity through the vascular smooth muscle KATP channel, thereby affecting acute pancreatitis and the associated lung injury [90]. Endogenous H_2_S blocks sulfur mustard (SM)-induced oxidative damage through the Nrf-2 pathway [91]. Inhibition of CSE indicated anti-inflammatory outcomes in a murine model of pancreatitis-induced lung injury [10]. Bhatia et al. described that a high dose of NaSH (10 mg/kg I.P.) initiated lung inflammation and histological injury in mice, and this inflammation reverted to the baseline in 6 h post-injection, indicating that lethal consequences are due to high sulfide concentrations by the H_2_S donor (NaHS), which were rapidly cleared [92]. The administration of NaHS or H_2_S-releasing ACS15 [93] as a pre-treatment (10–15 mg/kg) decreased inflammation in pancreatitis-induced ALIs [89,93]. The study design influenced the role of NaHS. The high dose of 10 mg/kg of the H_2_S donor NaHS caused ephemeral lung inflammation in healthy subjects. At the same time, its administration as a pre-treatment induced anti-inflammatory effects in successive pancreatitis-induced ALI.

#### 3.2.2. Inhalation-Induced Lung Injury

This refers to the degree of tracheal, bronchial and pulmonary parenchymal damage caused by various inhalation of harmful substances. Mild cases may only irritate cough and chest tightness; however, airway obstruction and pulmonary inflammation may also occur in severe cases. Even acute emphysema syndrome and multiple organ dysfunction syndromes (MODS) endanger the lives of patients. Burning is associated with a higher expression of CSE mRNA in the liver. Although H_2_S administration reduces tissue damage and inflammation, H_2_S donors exacerbate lung injuries caused by burns and smog inhalation in sheep [94,95]. On the other hand, treatment with Na₂S suppresses ALI caused by burns and smoke by attenuating iNOS expression, peroxynitrite formation, acute respiratory distress syndrome, nitro yield (lysine measurement), protein (oxidized protein carbonyl formation) and PARP-1 activity in vivo [94]. H_2_S biosynthesis inhibitors such as AOAA and the mitochondria-targeted H_2_S donor AP39 reduce intracellular and pulmonary plasma oxidative stress measured as MDA levels and cause organ infiltration into leukocytes (pulmonary MPO levels), pro-inflammatory and anti-inflammatory effects (circulating IL-6 and IL-10 levels), and liver and kidney dysfunction index (ALP and creatinine, respectively) [96]. Therapeutic administration of H_2_S biosynthesis the inhibitor PAG in mice with lung injuries caused by burns reduces systemic inflammatory non-MPO activity [91].

#### 3.2.3. Ventilator-Induced Lung Injury (VILI)

Although lung-protective ventilation strategies (LPVS) are regularly practiced for patients with ARDS, ventilator-induced lung injury (VILI) has received widespread attention as a common complication. H_2_S relieves VILI by decreasing autophagy and endoplasmic reticulum stress in L2 cells and rats by decreasing PERK, PERK phosphorylation and nuclear expression ATF4 after treatment with NaHS [73]. The cyclin strain initiates nuclear NF-ҝβ, MAPK, JNK, p65, p38, and ERK; whereas the ER stress inhibitor 4-PBA or NaHS suppress them. The H_2_S donor NaHS and inhaled H_2_S prevent ALI caused by ventilators [39]. H_2_S decreases the migration of neutrophils and the release of cytokines, thus exerting anti-inflammatory effects [97]. H_2_S limits lung injury due to ventilators by hindering ROS production via the PI3K/Akt signaling pathway [74]. Francis, R.C. et al. recommended systemic endovascular treatment with Na2S, which prevents ventilator-induced lung injury and lung glutathione depletion by activating Nrf-2-dependent antioxidant gene transcription [98].

### 3.3. H_2_S and Asthma

Asthma affects about 334 million individuals and has a high global death ratio [99]. Therefore, asthma is a leading international health, economic and social concern. However, asthma’s pathogenesis includes allergies, chronic airway inflammation, responsiveness, airway neuromodulator disorders, genetic mechanisms, respiratory viral infections, neural signal transduction mechanisms and airway remodeling, and is not fully understood. The H_2_S metabolism influences the physiology of the lung and the development of asthma. Condensed endogenous H_2_S levels caused by the decline in H_2_S-producing enzymes may start an infective asthma infection aspect [52]. Alternatively, another study shows that a high dose (300 ppm) of H_2_S diffuses into the bloodstream through the lung membrane and causes hypoxemia, vasodilation and vasoconstriction [100].

Further evidence reveals that H_2_S stimulates the mitochondria to produce superoxide, which is converted to hydrogen peroxide (H₂O₂) to mediate hypoxic vasoconstriction [51]. A previous experimental study in asthmatic patients also found that exogenous H_2_S inhibits cell propagation and IL-8 release by attenuating the phosphorylation of ERK1/2 and p38 [101]. Clinical trials showed a strong association between serum H_2_S levels and forced expiratory volume (FEV 1.0), and a negative correlation with sputum cell count and sputum neutrophil percentage in acute asthma patients [30]. Wang, P. et al. reported that oxidative stress and mitochondrial dysfunction are related to asthma’s progress and development [52]. Similarly, antioxidants decrease mitochondrial dysfunction and oxidative stress in asthma [102]. A previous study revealed that endogenous H_2_S reduces airway inflammation and renovation in rat asthma models [39].

Besides, asthmatic mice characterized by inflammation and ovalbumin (OVA) decreased H_2_S production and CSE expression. Similarly, exogenous administration of NaHS reduced inflammation, and decreased airway infiltration by the neutrophils and eosinophils. Furthermore, NaHS reduced OVA, which initiates lung iNOS activation, restricting airway alterations. These facts indicate that H_2_S formed from CSE acts as an anti-remodeling and anti-inflammatory mediator in asthma’s pathogenicity [36]. In stable asthmatic patients or patients with acute exacerbation, H_2_S level is lower in their serum. On the other hand, serum [52] or an exhaled air H_2_S level [103] showed a positive relationship with forced expiratory volume and a negative association with neutrophil count [38]. The same results were recorded in small children having asthma [102].

### 3.4. H_2_S and Lung Cancer

Lung cancer is one of the most prevalent malignities globally and is a prominent source of cancer-associated mortalities. Recent studies indicated that the expression of different H_2_S-producing enzymes in cancer cells of different tissue types is high, suggesting the gas’s potential in developing the disease [83]. Szczesny et al. showed that severe mitochondrial DNA damage in lung cancer cells is linked to H_2_S and that normal lung epithelial cells do not have elevated cell A549/DDP cells (compared with A549 cells) [104].

### 3.5. H_2_S and Pneumonia

Pneumonia is an inflammation of the terminal respiratory tract, alveoli and interstitial lungs caused by microbes, physical and chemical factors, drug allergies and immune damage. Depending on the type of pathogen, it can be divided into fungal pneumonia, bacterial pneumonia, mycoplasma pneumonia and viral pneumonia. However, bacterial pneumonia is the most common kind of pneumonia and is a paramount public contagious infection. H_2_S has pro-inflammatory effects in various inflammatory models [105,106,107]. In the inflammatory model, plasma H_2_S levels, tissue H_2_S synthesis activity and CSE expression increased. Some of the literature has stated the anti-inflammatory effects of H_2_S treatments such as using s-diclofenac, ATB-429 and H_2_S donors (NaHS, Lawson’s reagent, N-acetyl cysteine) in inflammation to produce anti-inflammatory activity [58,95,107,108]. Recent studies have also shown a biphasic dose–response effect of H_2_S in inflammation [109].

H_2_S has an anti-inflammatory effect in a dose-dependent manner on pulmonary inflammation [110]. It has been shown that supplementation with H_2_S or inhibition of iNOS-induced elevation of the GSH/GSSG ratio is a possible mechanism for defending the airways from oxidative stress and inflammatory lung disease [111]. Prophylactic and therapeutic use of NaHS reduced total cell growth induced by ozone, containing macrophages and neutrophils. This type of treatment also reduces cytokine levels in broncho-alveolar lavage fluid, including TNF-α, factor (CXC motif) ligand 1, IL-1β and IL-6 levels; inhibits them bronchially; attenuates the ozone-induced increase in total MDA in broncho-alveolar lavage fluid and reduces the ratio of condensed glutathione/oxidized glutathione in the lung. Besides, NaHS can block and reverse the phosphorylation of p38 MAPK and heat shock protein [103]. This shows that H_2_S might have protective and therapeutic significance in treating airway diseases based on oxidative stress.

In 2013, Aslami et al. reported that NaHS might promote ATP synthesis and mitochondrial biogenesis by protecting oxidative phosphorylation to reduce organ damage in pulmonary sepsis caused by pneumococci [112]. Another study suggested that H_2_S produced by *Streptococcus pneumoniae* causes hemolysis via the enzymatic activity of HapE (a protein similar to cysteine desulfurase) [113]. In respiratory syncytial virus (RSV), H_2_S was found to have an overall inhibitory effect on paramyxoviruses (for example, human metapneumovirus (hMPV) and Nipah virus (NiV)) [114]. Pediatric cystic fibrosis can be chemically active, anoxic and highly condensed due to H_2_S formation [115], and H_2_S can upregulate cytokine and chemokine production, and aggravates NF-κB activation by participating in systemic inflammatory sepsis [116,117]. NaHS protects rat lungs from inflammatory responses through hemorrhagic shock, inhibiting oxidative stress, and Fas/FasL apoptotic signaling pathways [107]. In summary, different doses of NaHS and downregulation in lung inflammation were achieved through a reduction in pro-inflammatory chemokines and adhesion molecules.

### 3.6. H_2_S and Pulmonary Edema

Pulmonary edema is caused by the accumulation of tissue fluid and the loss of the intrapulmonary tract. A considerable quantity of tissue fluid cannot be absorbed through the pulmonary lymphatic vessels and the pulmonary venous system quickly if it is extravagated from the pulmonary capillaries, and collects in the alveoli, interstitial lungs and small bronchi on the lungs [118,119]. Ventilation causes serious obstacles. The clinical manifestations include breathing difficulties, cyanosis, extreme dyspnea, paroxysmal coughing and excessive sweating with a large amount of white/pink foamy sputum and double-lung balanced wet voice coverage. Experiments have shown that inhibition of transepithelial Na+ transportation gives a mechanism that improves edema development in H_2_S-exposed lungs [120,121,122]. NaHS decreases airway inflammation remodeling and tobacco smoke-induced oxidative stress, and enhances emphysema and developmental hypertension [39]. These protective outcomes are connected with improved phosphorylation of Akt and hindering of the downregulation of antioxidant molecules.

### 3.7. H_2_S and Bronchiectasis

Bronchiectasis relates to the destruction of the bronchial wall muscles and elastic tissues affected by chronic suppurate inflammation and fibrosis of the bronchus and its adjacent lung tissues, resulting in bronchial deformation and continued expansion. Typical symptoms are chronic cough and repeated hemoptysis. There are two different perspectives on the effects of H_2_S on bronchiectasis. Firstly, it leads to bronchodilation via regulation of the K-ATP channel and β-adrenergic receptors [123]. Secondly, the relaxation effect of NaHS is inactivated by Ca2+ influx and cholinergic receptor blockade [124]. Bronchiectasis is associated with increased Cl^–^ and IκB phosphorylation. H_2_S regulates Cl^–^ levels and decreases phosphorylated IκB expression, inhibiting the upregulation of pro-inflammatory cytokines in epithelial airway cells [125].

### 3.8. H_2_S and Pulmonary Fibrosis

**Figure 4:**
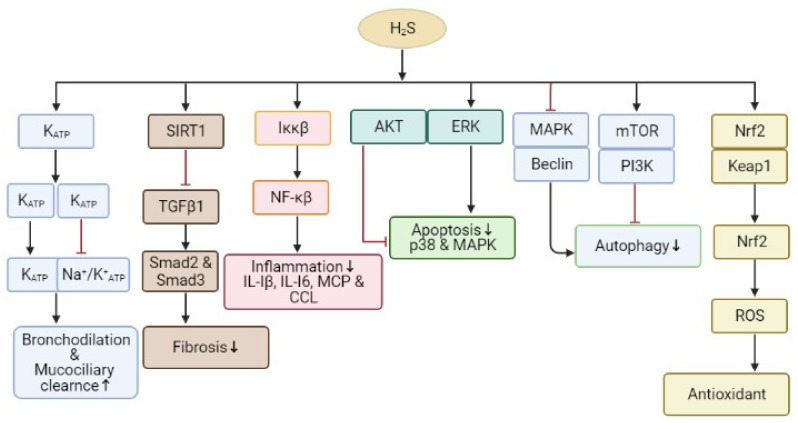
The signaling pathways underlying H_2_S regulation of inflammation, fibrosis, apoptosis, autophagy, antioxidant activity and bronchodilation. H_2_S has an anti-inflammatory outcome with diverse biological results, directly and indirectly decreasing activities such as Nrf2 activation. Abbreviations: ROS: reactive oxygen species, NF-ҝβ: nuclear factor-kappa B; Nrf2: nuclear factor erythroid-2 related factor 2; HO-1: heme oxygenase-1; PI3K: phosphoinositide 3-kinase; AMPK: AMP-activated protein kinase; ERK: extracellular signal-regulated kinase; TNF-α: tumor necrosis factor; TGF-β1: transforming growth factor-beta 1; Keap1: Kelch-like-ECH-associated protein; IL: interleukin; IKK: IҝB kinase.

Pulmonary fibrosis is the most common form of interstitial lung illness. It involves a slow exchange of normal lung parenchyma and fibrotic tissues, leading to an irreparable reduction in oxygen diffusion ability. The causes of pulmonary fibrosis are diverse, and there are many triggers, e.g., chemicals, allergens, radiation and environmental particles [126]. The anti-fibrotic effect of H_2_S on pulmonary fibrosis is that H_2_S protects against oxidative stress and inflammation [127]. Studies indicate that the H_2_S donor induces the nuclear buildup of Nrf-2 in lung tissues, thereby upregulating the expression of the Nrf-2-regulated antioxidant genes HO-1and Trx-1 in smoking rats [91,126]. Moreover, H_2_S can decrease cigarette smoke-induced inflammation by preventing ERK1/2, JNK and p38 MAPK phosphorylation, and adversely regulating NF-ҝβ activation, thereby preventing pulmonary fibrosis in smoking rats (Figure 4) [128]. Wang et al. also have shown that the anti-fibrotic effect of H_2_S relates to the inhibition of the TGFβ/Smad pathway [129]. In contrast, a high concentration of H_2_S (50–500 ppm) may produce occlusive bronchiolitis and pulmonary edema, leading to chronic inflammation and pulmonary fibrosis [130].

### 3.9. H_2_S and Sepsis

The incidence of sepsis is high, with more than 19 million severe sepsis cases occurring worldwide each year [131]. The underlying pathogenesis of sepsis remains unclear. It involves complex systemic inflammatory network effects, genetic polymorphisms, immune dysfunction and coagulopathy. H_2_S activates the selective transient receptor potential vanilloid 1 (TRPV1) by enhancing the upregulation of COX-2 and PGEM, coordinating with the neurogenic inflammatory response. The overproduction of substance P initiates a neuro-inflammatory process, namely ERK-NF-Κβ. ERK-NF-κB is activated in a TRPV1-dependent manner and significantly increases sepsis severity [90,132]. H_2_S upregulates substance P by activating the substance P receptor to coordinate the inflammatory response, leading to lung inflammation and sepsis damage [133]. The failure of exogenous H_2_S to prevent neutrophil migration caused a noteworthy decrease in mortality in a mouse model of ALI [90].

### 3.10. H_2_S and Lung Transplantation

Among the lung transplantation diseases, COPD, idiopathic pulmonary fibrosis (IPF), cystic fibrosis, sap-1 antitrypsin deficiency and idiopathic pulmonary hypertension are the main predisposing factors. In clinical practice, lung transplantation is a surgical process, either as single lung transplantation, double lung transplantation, cardiopulmonary transplantation or live lung transplantation. Lung ischemia reperfusion (IR) injury is still the main reason for the early mortality of lung transplantation [134]. A preliminary study reported an experimental model for the application of H_2_S inhalation after long-term ischemia. In this study, lungs pretreated with inhaled H_2_S showed an improvement in graft function during reperfusion, indicating the therapeutic use of H_2_S in the lung transplantation experimental model [135].

Similarly, pretreatment in a rat model with intra-peritoneal NaHS administration significantly improved pulmonary function, and decreased lipid peroxidation and MPO activity after lung transplantation. Besides, NaHS inhibits interleukin 1β but increases interleukin 10 levels in graft lung tissues [136]. The rat model of diabetes mellitus suffering from ischemia reperfusion after lung transplantation decreased ischemi-reperfusion-related oxidative stress after treatment with a slow-releasing H_2_S, GYY4137 [134]. H_2_S attenuated lung IR injury in Type 2 diabetic disorder through the initiation of lung SIRT1 signaling, which upregulates the Nrf2/HO-1 and eNOS-mediated antioxidant signaling pathways, therefore decreasing cell apoptosis and inflammation, and finally having a protective lung function.

### 3.11. H_2_S and Pulmonary Hypertension

Pulmonary hypertension (PH) is a chronic infection described by central pulmonary vascular pressure and may be caused by various disease processes. Regardless of the cause, PH is a progressive disease. New therapeutic drugs are often decompensated in the advanced stage and usually have a poor prognosis [137]. A decrease in the endogenous H_2_S pathway in hypertension and pulmonary vascular structural remodeling caused a high pulmonary blood flow in mice [138]. H_2_S inhibits arterial elastin expression in its extracellular matrix [35]. In a hypoxic rat model with pulmonary artery smooth muscle cells, H_2_S effectively inhibited a hypoxia-induced increase in cell proliferation, migration and oxidative stress in PASMCs [139]. H_2_S can enhance total antioxidant capacity by attenuating the GSSG content levels in hypoxia-induced pulmonary hypertensive rats’ lung tissue and exert an antioxidation effect [140]. Endogenous H_2_S is downregulated in PH, and pulmonary vascular remodeling is influenced through high pulmonary blood flow. NaHS and endogenous H_2_S can also prevent elevated pulmonary hypertension, pulmonary vascular remodeling and high pulmonary blood flow due to chronic hypoxia [39].

### 3.12. H_2_S and Sleep Apnea Syndrome (SAS)

According to the American Sleeping Society, SAS refers to the complete collapse of the upper airway, with the disappearance of airflow but the presence of respiratory motion, characterized by the airflow disappearing for more than 10 s, with significant chest breathing or esophageal pressure fluctuations. Central sleep apnea syndrome (CSAS) is characterized by the complete disappearance of airflow and respiratory movements that disappear for more than 10 s; the airway is not entirely blocked when ventilation is insufficient and airflow is weakened. Arousal and hypoxemia (>3% SaO_2_) occur frequently. Mice deficient in HO-2 produce the gaseous molecule carbon monoxide (CO) and exhibit sleep apnea, categorized through high apnea and hypopnea indices [141]. The glomus cells in the primary sensory organ, the carotid body (CB), are responsible for monitoring arterial blood O_2_, CO_2_ and pH levels. In rodents, obstruction of H_2_S production through CSE and pharmacologic or genetic methods inhibits carotid body activity, and hypertension is induced through intermittent hypoxia. During hypoxia, ROS triggers carbon monoxide synthesis by HO-2 and inhibits the synthesis of H_2_S by inhibiting CSE [142]. During hypoxia, as compared with normoxia, HO-2 produces less CO, subsequently augmenting the production of H_2_S, which motivates CB activity, resulting in increased respiration rate, heart rate and blood pressure. It has been reported that a decrease in CO and an increase in CB in H_2_S generation led to sleep apnea in HO-2 knockout mice and impulsively hypertensive mice [141].

### 3.13. H_2_S and Acute Respiratory Distress Syndrome

ARDS is a clinical disorder categorized by obstinate hypoxemia. It has attracted much attention due to its high mortality rate. The causes of acute respiratory distress syndrome are numerous, and the pathogenesis of ARDS caused by different reasons is also different. Oxidative stress, including the formation of superoxide (O2•−), might play a vital role in the pathogenesis of ARDS. H_2_S gas intoxication develops ARDS, demanding a high rate of percussive ventilation [143]. In addition to direct vasoconstriction, O2•− also reacts with NO to form peroxynitrite and other reactive nitrogen, effectively reducing NO bioavailability. H_2_S inhibited O2•− formation in porcine aorta-derived endothelial cells, and the adenylate cyclase-PKA pathway upregulated NADPH oxidase. H_2_S-donating sildenafil may effectively treat ARDS through increasing cAMP and preventing Type 5 phosphodiesterase activity [144].

### 3.14. H_2_S and Bronchopulmonary Dysplasia (BPD)

BPD is a chronic lung infection that causes persistent respiratory distress. It is caused mainly by hyperoxia, mechanical ventilation and inflammation, and categorized through impaired alveolar growth and complex pulmonary hypertension (PHT) [84,145]. It exhibits substantial streaks and overexpansion characteristics in X-rays. H_2_S showed a protective effect in a BPD rodent model through HO-1 [145]. GYY4137 preserved and restored mitochondrial function in alveolar epithelial cells and normal alveolar development in mice pups exposed to hyperoxia for 2 weeks after birth [84]. The effect of NaHS on the migration of alveolar Type II (ATII) cells was reduced by glibenclamide, implicating ion channels, and was accompanied by Akt activation, suggesting two probable mechanisms of H_2_S action. Such work triggers more study of H_2_S as an applicant interventional approach to bind the prevented alveolarization linked with BPD [146].

## 4. H_2_S in the Physiopathology of Airways

H_2_S regulates some airways’ physiological processes, both in human and animal models, as summarized in Table 1. Disorders of the endogenous formation of H_2_S are connected to pathological procedures and the development of numerous ailments, including hypertension, hypoxic pulmonary hypertension and myocardial injury [35,147,148]. H_2_S mediates smooth muscle relaxation via high airway activity inhibition caused by smoke from cigarettes, ozone and ovum albumin. On the other hand, H_2_S intensifies the said effects if inhibited [149]. This relaxation was due to endogenous H_2_S production in porcine airways [150]. The precursors of H_2_S, such as L-cysteine, also produce relaxation in the airway, but an inhibitor of CBS, amino oxy-acetic acid, inhibits the relaxation activity. The relaxation of smooth muscle also involves the inhibition of H_2_S, which relaxes the smooth muscle inhibition of Ca2+ release via InsP3 receptors [151] and the K+ channel [150]. Tracheal smooth muscle cells of mice showed hyper-movement through the potassium channels by stimulating the large-conductance calcium-activated potassium channel (BKCa) after treatment with NaHS. Such action causes the inhibition of Ca2+ influx and hyper-polarization of cells [152]. In contrast, relaxation was caused by H_2_S via opening the KATP channels in smooth muscle cells of human airways [70]. The inhibition of phosphorylation of extracellular p38MAPK and ERK1/2 by H_2_S has an inverse effect on the multiplication of smooth muscle cells and interleukin-8 release induced in fetal calf serum [101]. H_2_S also regulates the physiological function of vessels, thus acting as a vaso-relaxant agent [153]. H_2_S also enhances NO signaling in vessels [45] and vasodilatation of the pulmonary artery in rat [154].

## 5. H_2_S in Pulmonary Inflammation

Endogenous and exogenous H_2_S acts in the respiratory system via controlling mucolytic function. H_2_S can make the mucus less tacky, as it supports mucin cracking through connections with disulfide bonds [155]. H_2_S activates electrolyte absorption via the imitation of ATP-sensitive potassium channels (KATP) and prevents the Na+/K+-ATPase and calcium-sensitive potassium channels in human bronchiolar epithelia [156]. The function of exogenous H_2_S in lung ailments has been considered by using H_2_S donor representatives. The significance of slow or fast H_2_S-releasing elements in inflammatory reactions was generally evaluated by consuming molecules that are capable of producing H_2_S with deliberate and continuous discharge kinetics. Treatment with NaSH, “a fast releasing” H_2_S donor, encourages a significant provocative and inflammatory response in rats, as estimated through amplified MPO activity and the occurrence of leukocytes in the lungs [105].

Furthermore, the slow-releasing H_2_S elements such as GYY4137 produced anti-inflammatory effects in vivo and decreased pro-inflammatory cytokines (IL-I6, IL-Iβ, and TNF) in LPS-induced pulmonary inflammation in a mouse model. Similarly, treatment with GYY4137 produces noticeable antioxidant effects by reinstating the antioxidant enzymes catalase and SOD in lung tissues, strengthening the balance between reduced and oxidized GSH [157]. GYY4137 also reduced pro-inflammatory genes’ expression via moderating the initiation of NF-ҝβ and IFN regulatory factor-3 (IRF-3) [114]. Post-transcriptional NF-ҝβ is a new mark of H_2_S to reverse vascular inflammation. H_2_S blocked the initiation of the NF-ҝβ pathways in a model of nanoparticles. Pyrrole induced an inflammatory reaction in pulmonary artery endothelial cells via the sulfidation of IK-ҝβ of Cys179 residue, therefore preventing IK-ҝβ action. These types of process give clues about defending initiation against pulmonary vascular inflammation, pulmonary arterial hypertension and vascular modeling in vivo [158]. Moreover, treatment with GYY4137 prohibited lung injury and neutrophil migration, decreasing chemoattractant signaling molecules in vitro in the lung tissue of a mouse model of LPS-induced acute lung injury [157]. Remarkably, H_2_S moderates the entry of leukocytes from the bloodstream to swollen tissues [159], and this consequence depends on the initiation of annexin-1 pro-resolving pathways [160]. H_2_S considerably reduces pro-inflammatory cytokines such as IL-6 and IL-8 and augmented anti-inflammatory IL-10 in the plasma and lung. H_2_S directly repressed the pro-inflammatory reaction and ROS development in neutrophils, emphasizing the valuable prospective H_2_S donors as acute lung injury prophylactics (Figure 4). H_2_S promotes anti-inflammatory consequences via epigenetic changes.

H_2_S regulates the methylation and acetylation of histones, which governs the production of pro-inflammatory elements. Hence, H_2_S contributes to decreasing cytokine discharge and subsequent improvement of LPS in rats [155,157]. Treatment with diallyl disulfide (DADS) and arylthioamides as H_2_S donor induced a protective result in naphthalene-induced lung injury [161,162]. Therapy with DADS increases GSH levels in the lung tissue, preventing pro-inflammatory cytokine (IL-6, IL-8, and TNF) release relating to overcoming lung inflammatory cell deployment and precise neutrophil infiltration [163]. Sulforaphane, a naturally occurring isothiocyanate capable of generating H_2_S [164], reduced the release of pro-inflammatory mediators in a mouse model of LPS-induced acute lung injury. Sulforaphane mediates lung protection through transcription factor Nrf-2 by regulating mitochondrial function and energy use. Nrf-2 is accountable for inducing the expression of multiple antioxidant genes and averting oxidative injury. This kind of mechanism of action has also been defined for synthetic thiocyanate, whose H_2_S donor profile has been extensively discussed [147,165,166,167,168,169,170,171]. Anethiole dithiolethione has often been an H_2_S donor or a compound for developing H_2_S-releasing hybrid drugs with the non-steroidal anti-inflammatory agents H_2_S-diclofenac and H_2_S-aspirin. The previously mentioned new drugs possess anti-inflammatory outcomes compared with the “parent drugs” aspirin and diclofenac, showing their efficacy in decreasing lung MPO activity in a rat model of LPS-induced diclofenac-associated septic shock after H_2_S-diclofenac administration [172]. According to a recent study, PM considerably improved airway inflammation and emphysema in mice, calculated through the alveolar destruction index, total cell pro-inflammatory cytokinesis (IL-6, IL-8 and TNF), neutrophil counts and CXCL1 broncho-alveolar lavage fluid. H_2_S decreased particulate matter (PM)-induced mouse emphysema and airway inflammation by decreasing oxidative stress as assessed by 8-OHdG concentration in lung tissues. H_2_S plays a protective role in PM-induced rat emphysema and airway inflammation by preventing NLRP3 inflammasome development and apoptosis produced through fine particulate matter(pM2.5) contact with A549 cells but not in Nrf2-silenced cells [173].

**Table 1:** Pathophysiological actions of H_2_S in the lung.

Action	H_2_S	References
Vasodilation	↑	[153,154]
Stable asthma	↓	[35,36,38,52,68,102,174]
Bronchodilation	↑	[103]
Angiogenic activity	↑	[22]
Pro-inflammatory action	↑	[61]
Anti-inflammatory action	↑	[61]
Airway hyper-reactivity	↑	[63,64]
Asthma exacerbation	↓	[35,36,38,52,68,102,174]
Stable COPD	↑	[65]
COPD exacerbation	↓	[37]

↑ = Increased, ↓ = decreased.

## 6. Clinical Trials of H_2_S Donors

Ik-1001(Na2S) was the first compound chosen to administer as an H_2_S donor in a clinical trial in 2009. Directed IK-1001 (NCT00879645) was the first clinical trial conducted, which was soon terminated, as it was incapable of reducing the sulfide level. Not being capable to consistently measure sulfide is a serious issue for a compound’s approval. The main concerns raised by the scientific community were because of the highly volatile and quickly absorbed nature of exogenous sulfide [174]. Numerous sulfide compounds are present in biological systems, and sulfide participates in several chemical processes [175], which reveals that these endogenous compounds are highly dynamic.

In contrast, the exogenous administration of H_2_S might result in the equilibrium of this entire system in ways that we have not wholly known until now. After that, IK-1001(NCT00858936), during secondary trials, affected coronary artery bypass and stopped with this issue. A later clinical trial was established using ST-elevation myocardial infarction (NCT010074610), which was also stopped due to safety issues. Therefore, an aqueous solution of liquid H_2_S called IK-1001 can be typically administered with H_2_S-releasing salts or inhaled H_2_S. Neither administration of H_2_S through inhalation nor injection of H_2_S donors will possibly be utilized in the clinical trials due to airway mucosal injury. Still, there is the possibility of poisonous sulfide concentrations eventually being produced [174]. Inhalation of 330 ppm H_2_S via sub-lethal administration has been used as a model to study lung injury [86,176]. Efforts to evade the airway inflammation of gaseous H_2_S were applied to an extracorporeal membrane for lung ventilation in a pre-clinical study. However, there was limited development in the effect of cardiopulmonary bypass [100]. The trial utilized a combination of organic sulfide-releasing compounds and salts (named SG-1002(NCT01989208), which has been used in heart failure studies. This trial proved to be safe and well-tolerated in Phase 1 trials; however, it failed in the Phase II (NCT02278276) trial. An excellent prospect for H_2_S-based treatments is the reassessment of H_2_S donors or compounds that are now clinically permitted and have only recently been recognized to be capable of releasing H_2_S, such as sodium thiosulfate (STS) [177,178], which helped in cyanide detoxification and cisplatin overdosage (ammonium tetra-thiomolybdate (ATTM) [179,180], and is allowed for Wilson’s disorder (a copper metabolism ailment), and zofenopril [181], an inhibitor of angiotensin-altering enzymes permitted for hypertension. These compounds all have been verified widely and are recognized to have worthy safety profiles. For example, Dyson et al. revealed that ATTM led to a 50% decrease in infarct size in mouse models of myocardia and cerebral I/R in addition to the improved persistence of later hemorrhage [180].

The good wellbeing profile of STS [182], specifically, might be connected to the circumstance that thiosulfate itself is an endogenous intermediate of oxidative H_2_S metabolism [177] and is recommended as a molecule with valuable H_2_S results [183], specifically in hypoxic circumstances [177]. The clinical trial of IK-1001 in renal injury utilized thiosulfate as an unintended measure of H_2_S release by their compound, though this was finally found to be ineffective. STS is presently in a Phase II clinical trial to preserve cardiac function in SREMI. Concerning the lung, as revealed before, STS was helpful in murine models of intratracheal LPS and CLP [80]. While Sakaguchi et al.’s results supported these effects, they determined a practical consequence of STS in the lung, i.e., enhanced gas altercation and lung processes in an interpreter-related large animal model of hemorrhagic shock. Therefore, STS is a compound providing hope for the advancement of therapeutic H_2_S administration in ALI in clinical settings [80].

In animal experiments, NSAIDs conjugated to H_2_S (for example, celecoxib and naproxen) revealed a robust protective effect on gastrointestinal epithelium as matched with the parent’s lethal drug results [184]. For example, the H_2_S-releasing naproxen known as ATB-346, which releases H_2_S through a hydrolytic mechanism [184], was confirmed to have better anti-inflammatory results in animal models, decreasing leukocyte migration and decreasing TNF-α and TNF-αβ expression [184,185,186]. Another H_2_S donor is S-mesalamine (ATB-429), which is utilized for the treatment of inflammatory colitis. ATB-429 played a protective role in the gastrointestinal mucosa and had more remarkable anti-inflammatory outcomes than the parent drug [106]. Hence, ATB-429 might be a worthy applicant for decreasing inflammation [187]. Correspondingly, NBS-1120 had an excellent protective role in an animal model of inflammation compared with aspirin [188]. While GYY4137 was show to directly obstruct inflammation in a mouse model by inhibiting different inflammatory molecules [189], GYY4137 can reduce LPS-evoked septic shock [190]. These previously discussed studies emphasized the curative prospects of H_2_S donors for the treatment of inflammation and respiratory diseases. However, extra in vivo analyses and studies are necessary to endorse the effectiveness of these H_2_S donors, their safety, and their possible use in such diseases.

## 7. Perceptions, Limitations and Prospects

H_2_S executes a broad range of pathophysiological functions, including vasodilatation to lower the blood pressure, initiation of angiogenesis, signal regulation of neuronal action and regulation of glucose homeostasis, which have been widely demonstrated beside NO and CO. H_2_S was previously considered an environmental contaminant but is now widely recognized as an important biological and pharmacological medium, and is considered to be the third endogenous gas transmitter in mammals, There is increasing confirmation that H_2_S plays a crucial part in respiratory diseases, revealing that the metabolic machinery and mechanisms of H_2_S are an essential research topic in respiratory diseases. H_2_S is mainly metabolized by CSE, CBS and 3-MST in mammals. The metabolic pathway of H_2_S is different in different organs and tissues. There is increasing evidence that H_2_S plays a crucial role in respiratory diseases. Investigating the metabolic machinery and mechanisms of H_2_S in respiratory diseases is an important research topic that may help develop new drugs. The scientific relationships of H_2_S in mammals should be widely observed and studied experimentally to elucidate the expression and function of H_2_S-producing enzymes in different organs and tissues, and provide new ideas for the better development of new H_2_S donors and targeted clinical therapies.

Respiratory disorders are general and often-occurring ailments with a relatively high mortality rate. The primary lesions are in the trachea, bronchus, lungs and chest. In addition to the existing pathophysiological mechanisms, further research into and clarification of the new underlying mechanisms and new signaling pathways associated with respiratory diseases are needed. At present, some progress has been made by using animal models to study the molecular mechanism of H_2_S in respiratory injury. The molecular targets of H_2_S in the respiratory system also require further investigation. Because altered amounts of H_2_S-releasing compounds may produce various therapeutic outcomes, further appropriate dose ranges should be studied to achieve better therapeutic results. Moreover, new H_2_S-releasing donors should be designed and identified to increase the therapeutic effect by mediating H_2_S concentrations in human disease, and whether this effect would reduce long-term disease and mortality.

The present evidence proposes that H_2_S has a function in regulating and maintaining vital biological progressions in animals. Despite the noteworthy development of H_2_S donors, there is still an absence of compounds that can address all the requirements for the perfect H_2_S donor in clinical studies. There are major gaps in our understanding that obstruct the clinical usage of H_2_S donors. Many questions need to be answered, such as (i) what the H_2_S-releasing compounds are, (ii) the therapeutic concentrations of H_2_S and its compounds, (iii) the concentrations at which H_2_S and its donors become toxic, (iv) the level of toxicity, (v) the mechanisms of H_2_S release from the H_2_S donors and drugs, (vi) the administration of H_2_S in vivo at a constant rate, (vii) the mechanism action of H_2_S, (viii) the monitoring of plasma levels of H_2_S and its products, (ix) what the differences between H_2_S administration in vivo and in vitro are, (x) assessing the sensitivity and specificity of H_2_S, (xi) the selection of patients for assessing the effectiveness of H_2_S drugs, and (xii) finding appropriate doses of H_2_S or its donors for treatment over a reasonable period in respiratory disease, and in pre-clinical and clinical studies. In conclusion, a deeper understanding of the exact molecular mechanisms behind the role of H_2_S in the development, progression, prevention and treatment of respiratory diseases is important for using appropriate doses of H_2_S or its donors to improve its clinical efficacy.

## Abbreviations

H_2_S	hydrogen sulfide
CBS	cystathionine beta-synthase
CSE	cystathionine γ-lyase
3-MPST	3-mercaptopyruavte sulfurtransferase
COPD	chronic obstructive pulmonary disease
SQR	sulfide-quinone reductase
TST	thiosulfate sulfurtransferase
CAT	cysteine aminotransferase
GSSH	glutathione
SMC	smooth muscle cells
ROS	reactive oxygen species
ALI	acute lung injury
NO	nitric oxide
ATF-30	transcription factor-3
OA	oleic acid
ECM	extracellular matrix
MLC	myosin light chain
PHT	pulmonary hypertension
SM	sulfur mustard
NSAID	non-steroidal anti-inflammatory drugs
MODS	multiple organ dysfunction syndrome
MDA	malondialdehyde
MPO	myeloperoxidase
ARDS	acute respiratory distress syndrome
LPVS	lung-protective ventilation strategies
VILI	ventilator-induced lung injury
FEV	forced expiratory volume
LTx	lung transplantation
PH	pulmonary hypertension
RSV	respiratory syncytial virus
IPF	idiopathic pulmonary fibrosis
CSAS	central sleep apnea syndrome
SAS	sleep apnea syndrome
hMPV	human metapneumovirus
CB	carotid body
CO	carbon monoxide
BPD	bronchopulmonary dysplasia
ATII	alveolar Type II
NaHS	sodium hydrosulfide
HO-1	heme oxygenase-1
STAT-3	signal transducer and activator of transporter-1
Nrf-2	nuclear factor erythroid-2 related factor
NF-қβ	nuclear factor-kappa B
PI3K	phosphoinositide 3-kinase
ERK	extracellular signal-regulated kinase
AMPK	AMP-activated protein kinase
TNF-α	tumor necrosis factor-α
TGF-β1	transforming growth factor beta 1
SOD	superoxide dismutase
IL	interleukin
IKK	IқB kinase
Keap1	Kelch-like-ECH-associated protein
ADT-OH	5-(hydroxyphenyl)-3H-1:2-dithiole-3-thione
ATB-429	4-(5-sulfanylidenedithiol-3-y) phenyl 5-amino-2-hydroxybenzoate
STS	sodium thiosulfate
